# Supplementary material for: Transcriptomic and Metabolic Profiling of High-Temperature Treated Storage Roots Reveals the Mechanism of Saccharification in Sweetpotato (Ipomoea batatas (L.) Lam.)
Source: Int J Mol Sci. 2021 Jun 22;22(13):6641. doi: 10.3390/ijms22136641 (PMC8267658; doi:10.3390/ijms22136641)
Supplement: Supplementary file 1 [file ijms-22-06641-s001.zip › ijms-1245680-supplementary.pdf]

Supplementary Information

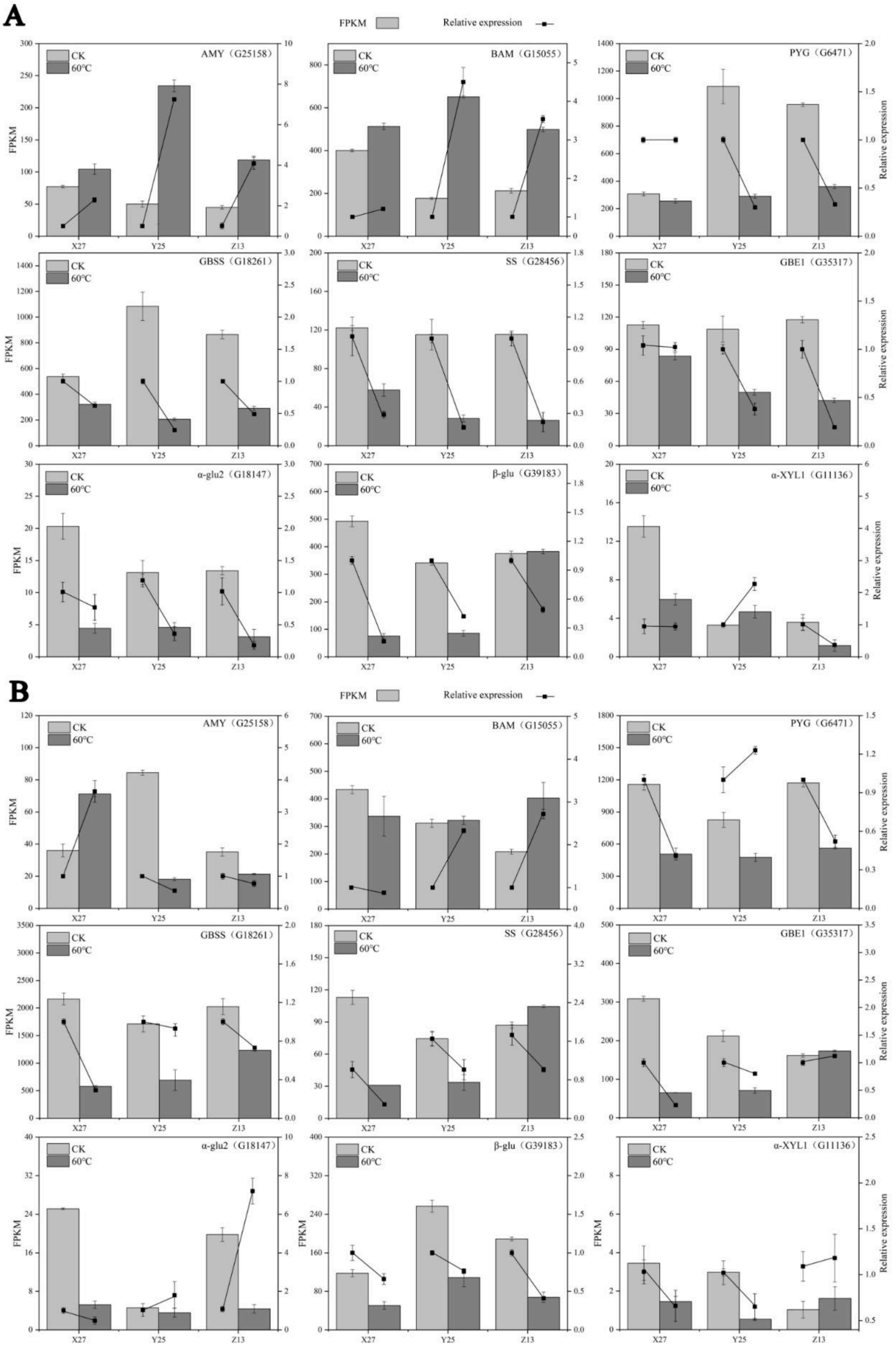

Fig. S1 qRT-PCR validation of genes associated with maltose metabolism in Sweetpotato samples at different growth period. Nine differentially expressed genes were selected for qRT-PCR analysis at different stages, including *AMY*, *BAM*, *PYG*, *GBSS*, *SS*, *GBE1*, *α-glu*2, *β-glu* and *α-XYL1*. Figure A and B are the data of sample determination at the growth period of 90 days(S1) and 130 days(S2), respectively.The qRT-PCR expression levels were calculated as a ratio relative to the level of expression in three samples CK samples of different growth period, which was set as 1. All qRT-PCR calculations of expression were normalized using the Ct value corresponding to *IbARF* gene. Values are the means±SE of three biological replicates.

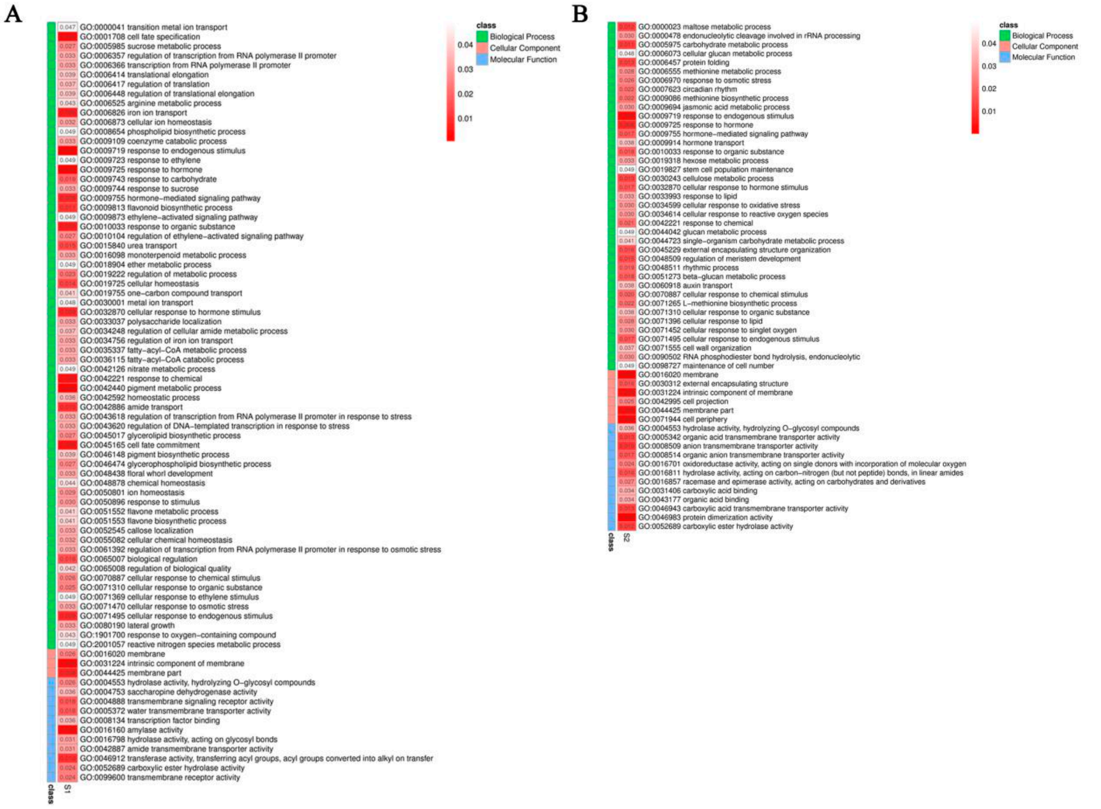

Fig. S2 Go annotation analysis of samples from S1and S2 growth stages A&B. Functional classification of DEGs enriched by GO annotation in sweetpotato for S1 and S2, respectively. The darker the red in the picture, the more significant the metabolic pathway.

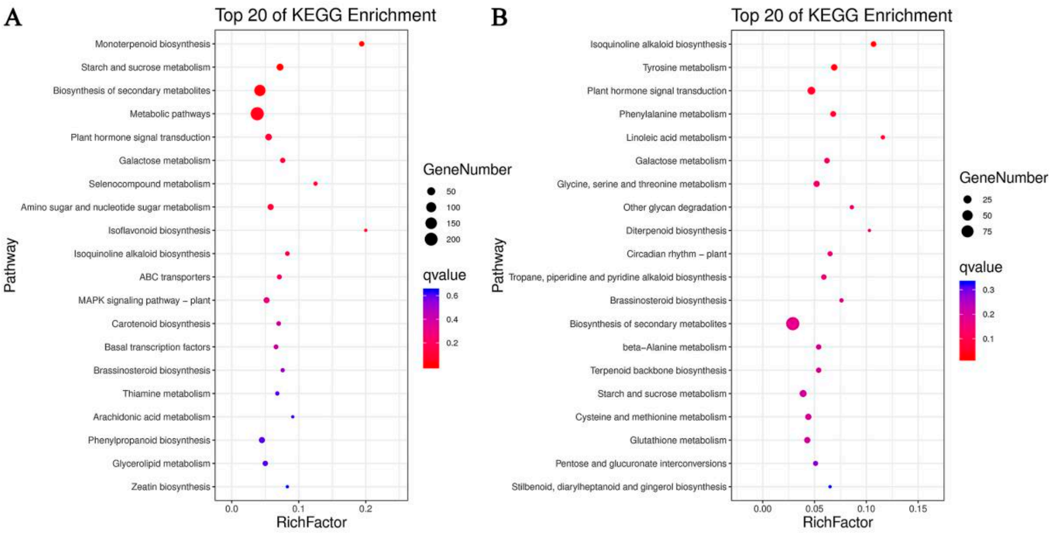

Fig. S3 KEGG pathway analysis of samples from S1and S2 growth stages A&B. The statistics of the number of DEGs in different metabolic pathways were obtained by KEGG enrichment analysis in sweetpotato for S1 and S2, respectively.

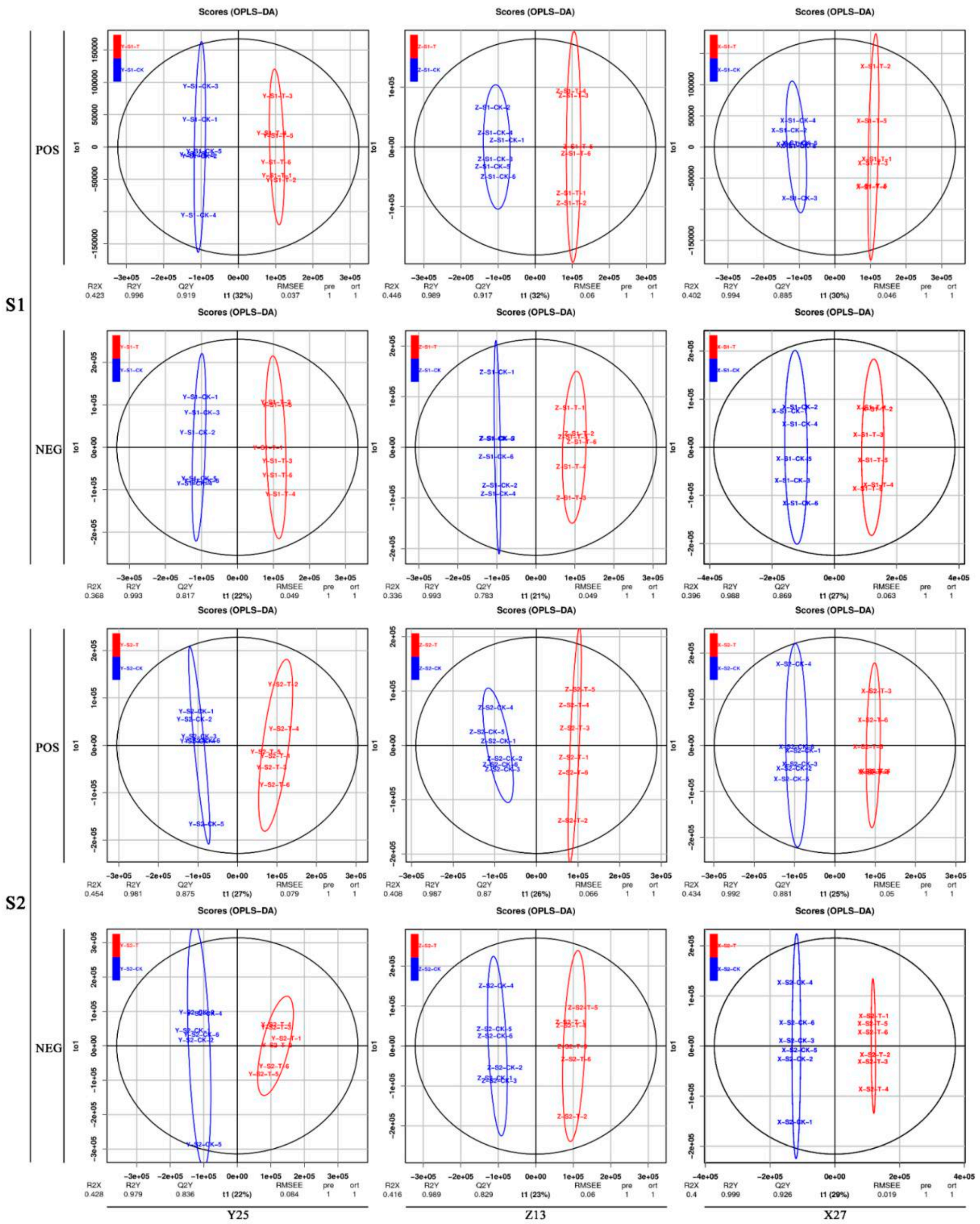

Fig. S4 (O)PLS-DA score plot of sweetpotato samples at different growth period R2X, R2Y and Q2 are used to evaluate the (O) PLS-DA evaluation model. The closer the three indexes are to 1, the more stable and reliable the model is. When Q2 > 0.5, the prediction ability of the model is better, and when Q2 > 0.9, the prediction ability of the model is excellent.

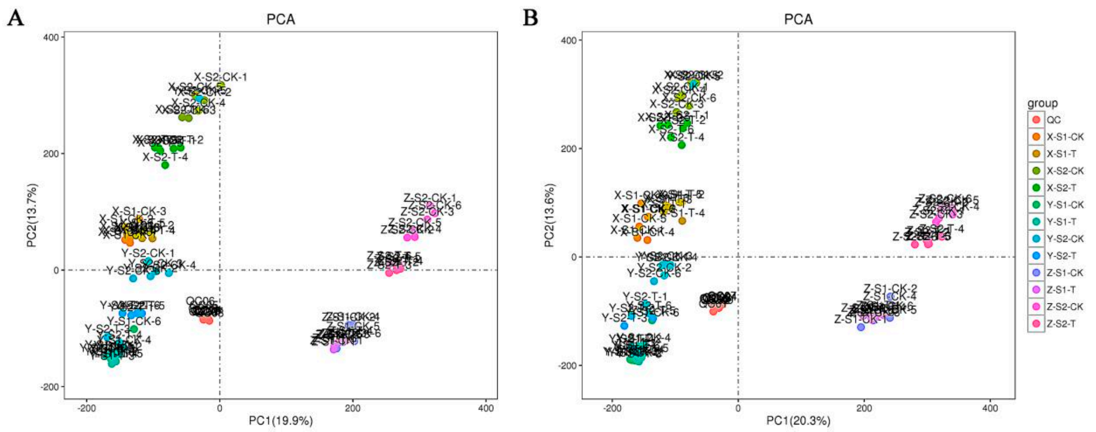

Fig. S5 Principal component analysis (PCA)of metabolome sample relationship Figures A and B represent the principal component analysis of the sample relationship in the determination of cation and anion channels, respectively.

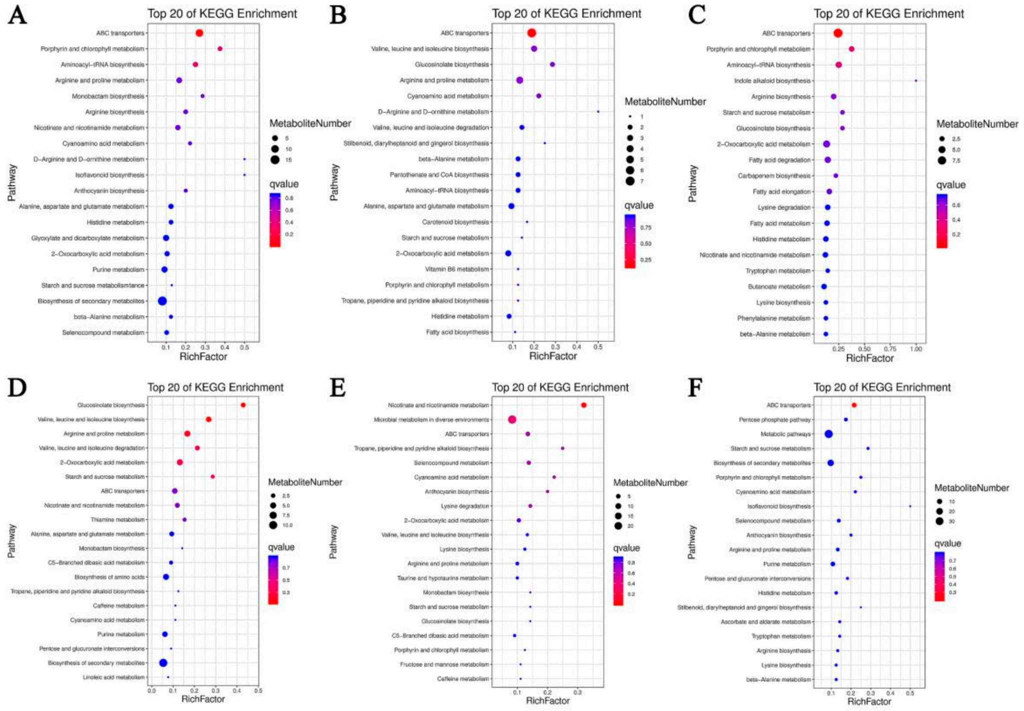

Fig. S6 The first 20 metabolic pathways obtained by KEEG enrichment analysis A,B&C. The first 20 metabolic pathways enriched by DAMs of sweetpotato cultivars Y25, Z13 and X27 during the growth stage S1. D,E&F. The first 20 metabolic pathways enriched by the DAMs obtained from three sweetpotato cultivars Y25, Z13 and X27 during the growth stage S2, respectively.

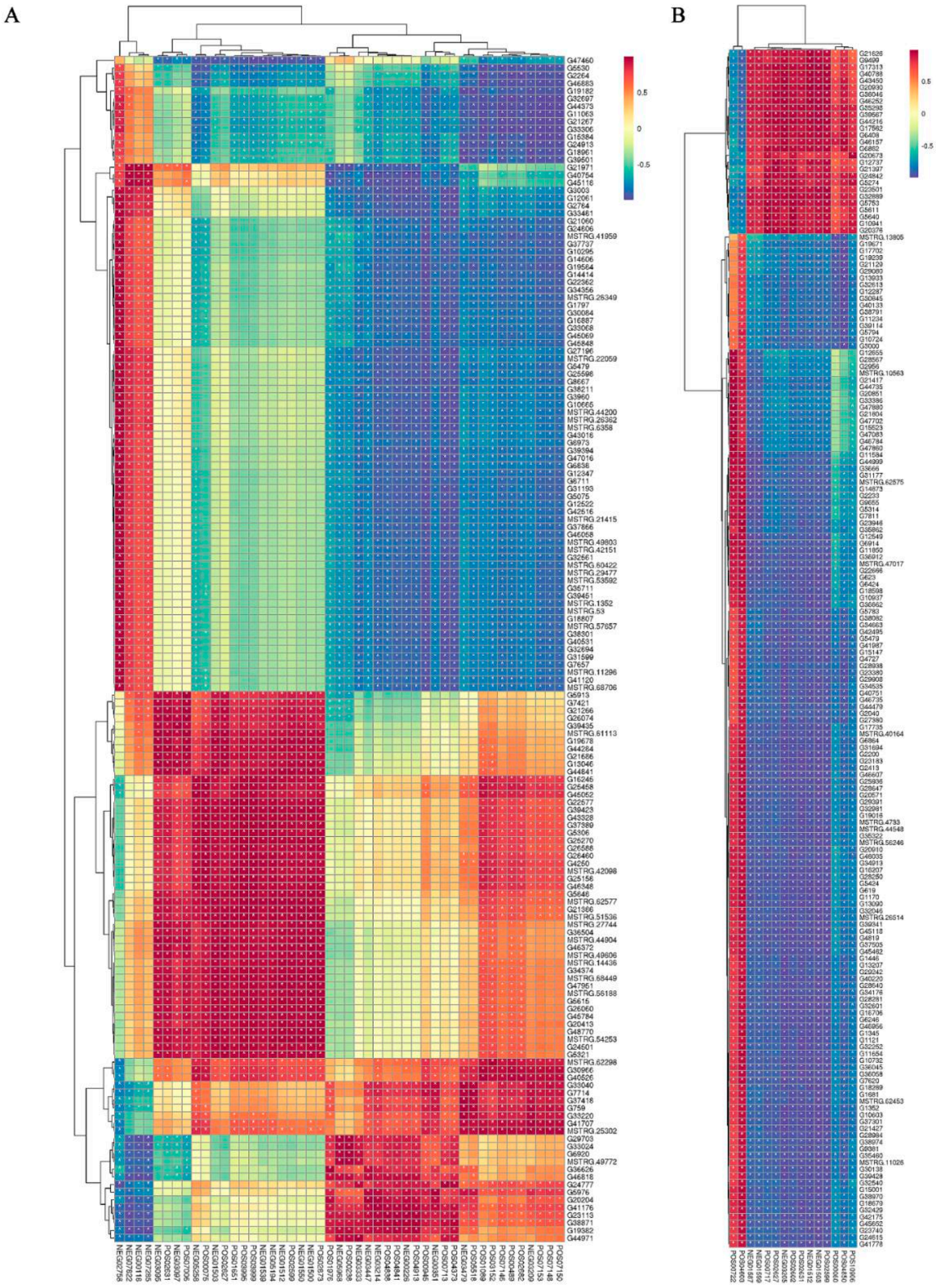

Fig. S7 Correlation analysis of between DEGs related to sugar metabolism and DEMs Figures A and B represent the correlation analysis of between DEGs related to sugar metabolism and DAMs in S1 and S2 periods, respectively.

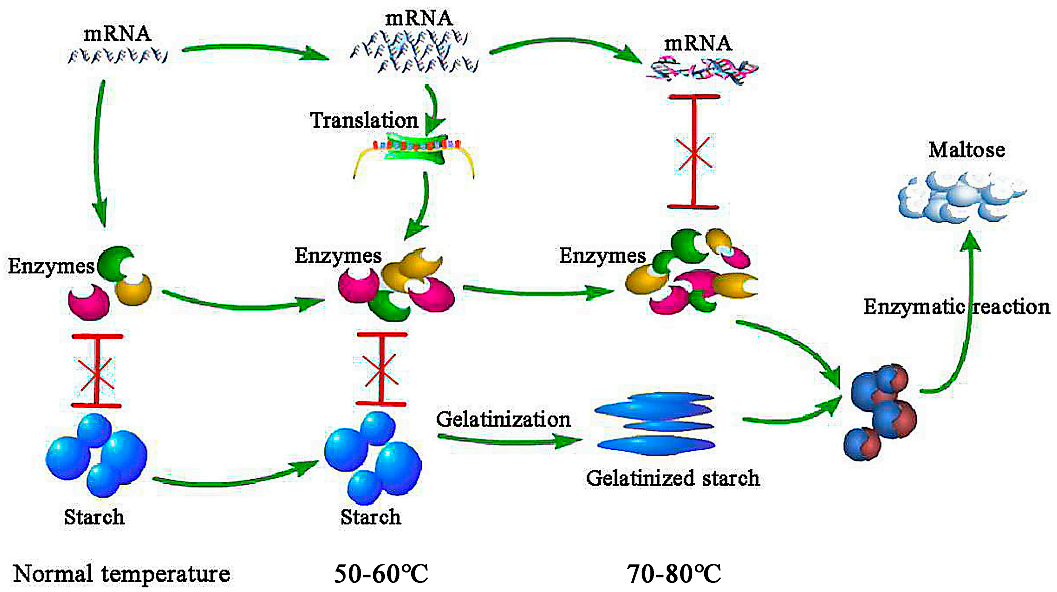

Fig. S8 Schematic diagram of saccharification process of sweetpotato storage root at high temperature

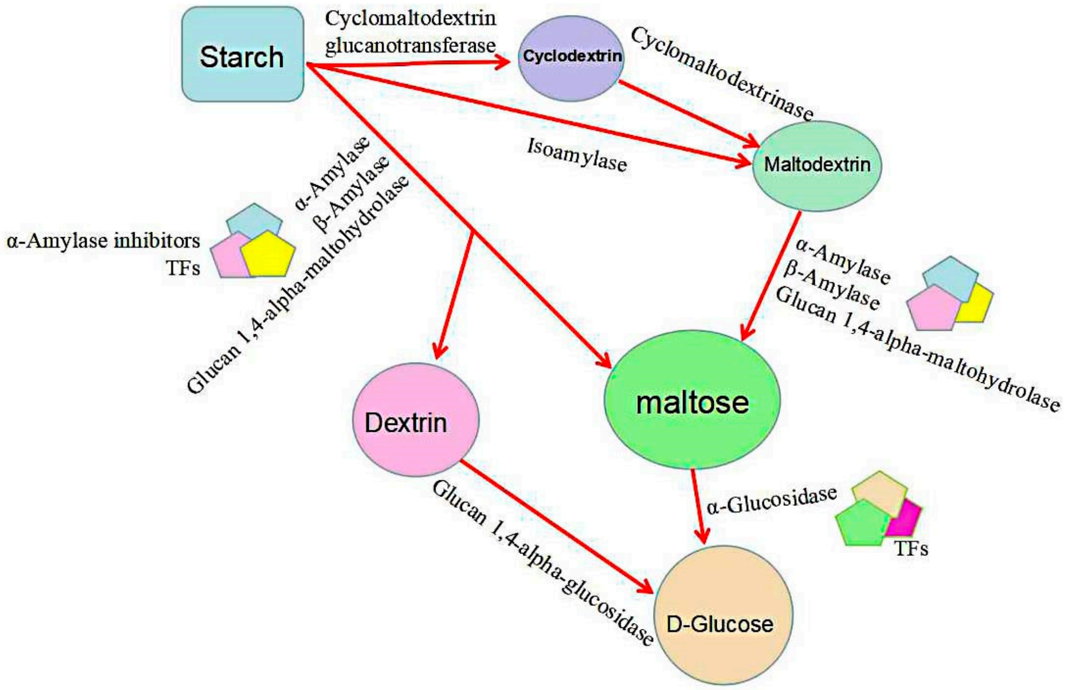

Fig. S9 Schematic diagram of key enzymes and regulatory factors in maltose synthesis pathway

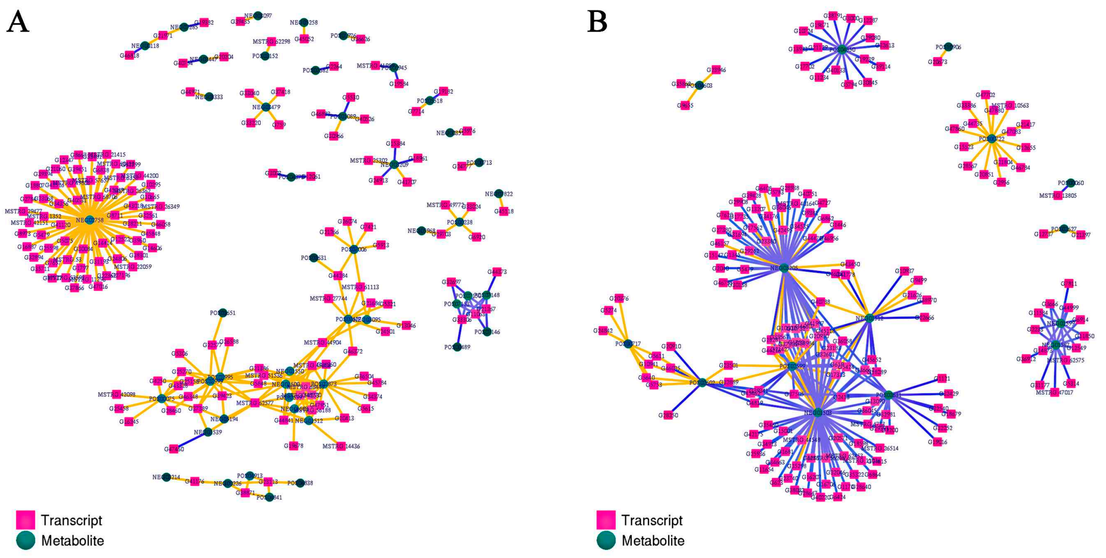

Fig. S10 Network analysis of DEGs and DAMs A&B. Network association of DEGs and DAMs in S1 and S2, respectively.

Table S1 qRT-PCR primers for validation experiment

Unigenes code	Direction	Sequence (5'->3')	Length
JX177359	Forward	CTTTGCCAAGAAGGAGATGC	20
	Reverse	TCTTGTCCTGACCACCAACA	20
G25158	Forward	AGCAGCAAGGTGGATGGTAC	20
	Reverse	GCCTTTATCCCTTTGCCGTG	20
G15055	Forward	GGTTGGACCACTCCATACGG	20
	Reverse	GTGTTGTAGTAGCCTGCGGT	20
G6471	Forward	CACTGAACAAGCTTGGCCAC	20
	Reverse	AGCCAATCTTCAGCCACCTC	20
G18261	Forward	AGCCAAGCTGTGACTCACAA	20
	Reverse	AGTCCACCAGTTTTGCACCA	20
G28456	Forward	CGAGTAAGAGCGGCGGATAA	20
	Reverse	AAACTGCCGTCCCCATCTTT	20
G35317	Forward	TGGTATGCCTGGACTTTGCC	20
	Reverse	CTGGTCATGGCTCTCTGCAT	20
G18147	Forward	GCTTTGAAACAAGCGACCGT	20
	Reverse	TGAAGCAGAATGGGCTGGAG	20
G39183	Forward	GTGCCCTGGGGAATGTACAA	20
	Reverse	CGCGTGGTAGCCTATCACAT	20
G11136	Forward	GGAATGTGAATGGGGAGGCA	20
	Reverse	ACCAATAGGGCATTGCAGCT	20

Table S2 Base quality analysis RawData(bp):total number of bases in RawData; BF: number of bases before filter; AF: number of bases after filter; Q20 (%): the number of bases whose sequencing base quality value reached above Q20 level and its percentage in RawData (or CleanData); Q30 (%): the number of bases whose sequencing base quality value reached above Q30 level and the percentage in RawData (or CleanData); N (%): the number of N bases in single-ended read and the percentage of N bases in RawData (or CleanData); GC (%): the proportion of sequence base GC before and after filter.

Sample	RawData(bp)	BF Q20(%)	BF Q30(%)	BF N(%)	BF GC(%)	CleanData(bp)	AF Q20(%)	AF Q30(%)	AF N(%)	AF GC(%)
X-S1-CK-1	7610918100	7407503216 (97.33%)	7067060620 (92.85%)	1041233 (0.01%)	3709323843 (48.74%)	7561040017	7364480762 (97.40%)	7027842665 (92.95%)	251745 (0.00%)	3683642830 (48.72%)
X-S1-CK-2	5807200500	5655435792 (97.39%)	5399497459 (92.98%)	805481 (0.01%)	2840924722 (48.92%)	5759430164	5613261201 (97.46%)	5360745306 (93.08%)	192765 (0.00%)	2815907983 (48.89%)
X-S1-CK-3	6207518700	6038579949 (97.28%)	5756814466 (92.74%)	849571 (0.01%)	3019575670 (48.64%)	6164845320	6001803471 (97.36%)	5723280817 (92.84%)	204350 (0.00%)	2997872069 (48.63%)
Y-S1-CK-1	7003012200	6815195296 (97.32%)	6500291470 (92.82%)	970026 (0.01%)	3475345086 (49.63%)	6949785161	6768509323 (97.39%)	6457462164 (92.92%)	231534 (0.00%)	3448308793 (49.62%)
Y-S1-CK-2	6071342400	5916801350 (97.45%)	5649130194 (93.05%)	819647 (0.01%)	3081962554 (50.76%)	6017928519	5868869388 (97.52%)	5604836069 (93.14%)	199087 (0.00%)	3054694891 (50.76%)
Y-S1-CK-3	6221026800	6051547341 (97.28%)	5770066131 (92.75%)	852212 (0.01%)	3081046427 (49.53%)	6177888192	6014021306 (97.35%)	5735757475 (92.84%)	205446 (0.00%)	3059548297 (49.52%)
Z-S1-CK-1	5970907200	5810272216 (97.31%)	5541570250 (92.81%)	817321 (0.01%)	2965985436 (49.67%)	5927376898	5772131024 (97.38%)	5506574023 (92.90%)	196574 (0.00%)	2943962466 (49.67%)
Z-S1-CK-2	5845753500	5690034417 (97.34%)	5428805491 (92.87%)	803356 (0.01%)	2916003877 (49.88%)	5803579311	5653231979 (97.41%)	5395158096 (92.96%)	194317 (0.00%)	2894461638 (49.87%)
Z-S1-CK-3	6515297700	6344084107 (97.37%)	6054200231 (92.92%)	892981 (0.01%)	3227980855 (49.54%)	6473460369	6307693683 (97.44%)	6020894322 (93.01%)	216962 (0.00%)	3206754811 (49.54%)
X-S1-T-1	5836394400	5670814667 (97.16%)	5398117848 (92.49%)	774754 (0.01%)	2893286961 (49.57%)	5776400479	5618551630 (97.27%)	5350447053 (92.63%)	185417 (0.00%)	2862500089 (49.56%)
X-S1-T-2	6586307400	6407600512 (97.29%)	6107272565 (92.73%)	870105 (0.01%)	3237912341 (49.16%)	6530711667	6359632068 (97.38%)	6063564864 (92.85%)	209485 (0.00%)	3209633725 (49.15%)
X-S1-T-3	7519417500	7296477686 (97.04%)	6934104787 (92.22%)	1002412 (0.01%)	3679766412 (48.94%)	7463597140	7249905946 (97.14%)	6892289464 (92.35%)	239225 (0.00%)	3651331787 (48.92%)
Y-S1-T-1	7476331800	7332187392 (98.07%)	7050493573 (94.30%)	301610 (0.00%)	3728583210 (49.87%)	7438327544	7299513405 (98.13%)	7020751920 (94.39%)	228443 (0.00%)	3708901708 (49.86%)
Y-S1-T-2	6070548300	5931180178 (97.70%)	5674253786 (93.47%)	31753 (0.00%)	2997670218 (49.38%)	6042680923	5908024137 (97.77%)	5653352412 (93.56%)	26061 (0.00%)	2983234682 (49.37%)
Y-S1-T-3	6296399700	6110981409 (97.06%)	5719911612 (90.84%)	49525 (0.00%)	3152418982 (50.07%)	6257910308	6077452293 (97.12%)	5690236205 (90.93%)	46990 (0.00%)	3132368504 (50.05%)
Z-S1-T-1	6257887800	6099568918 (97.47%)	5820551007 (93.01%)	45952 (0.00%)	3131086224 (50.03%)	6223117755	6070093324 (97.54%)	5793762865 (93.10%)	31133 (0.00%)	3112944723 (50.02%)
Z-S1-T-2	5589707400	5444811408 (97.41%)	5190133490 (92.85%)	37096 (0.00%)	2773649703 (49.62%)	5543929727	5404658991 (97.49%)	5153255304 (92.95%)	26622 (0.00%)	2749739518 (49.60%)

Z-S1-T-3	7016900700	6846495927 (97.57%)	6540970515 (93.22%)	51848 (0.00%)	3480046099 (49.60%)	6965902974	6801990302 (97.65%)	6500151073 (93.31%)	35739 (0.00%)	3453261072 (49.57%)
X-S2-CK-1	6684673500	6540346735 (97.84%)	6265391288 (93.73%)	35682 (0.00%)	3216052059 (48.11%)	6640689450	6501305233 (97.90%)	6229296532 (93.80%)	29366 (0.00%)	3192627416 (48.08%)
X-S2-CK-2	7946695800	7728993548 (97.26%)	7368329947 (92.72%)	1081619 (0.01%)	3812182339 (47.97%)	7875596407	7666439690 (97.34%)	7311018279 (92.83%)	260091 (0.00%)	3775267530 (47.94%)
X-S2-CK-3	7885152900	7675596835 (97.34%)	7323674413 (92.88%)	1089492 (0.01%)	3758886792 (47.67%)	7835630954	7633075179 (97.41%)	7284986365 (92.97%)	260938 (0.00%)	3733632117 (47.65%)
Y-S2-CK-1	6403725600	6241708488 (97.47%)	5961194113 (93.09%)	873393 (0.01%)	3149557489 (49.18%)	6346626731	6190533746 (97.54%)	5913974996 (93.18%)	211671 (0.00%)	3119979732 (49.16%)
Y-S2-CK-2	6197925300	6026762955 (97.24%)	5745152607 (92.69%)	850321 (0.01%)	2974951069 (48.00%)	6152423142	5987360079 (97.32%)	5709253859 (92.80%)	206669 (0.00%)	2951958114 (47.98%)
Y-S2-CK-3	6937801200	6751984488 (97.32%)	6441682623 (92.85%)	957830 (0.01%)	3345178165 (48.22%)	6890383150	6710854245 (97.39%)	6404113770 (92.94%)	228524 (0.00%)	3320922591 (48.20%)
Z-S2-CK-1	6214231200	6046046074 (97.29%)	5767193577 (92.81%)	855214 (0.01%)	3074681579 (49.48%)	6162787472	6000883579 (97.37%)	5725893011 (92.91%)	204605 (0.00%)	3047993494 (49.46%)
Z-S2-CK-2	7606427700	7401306824 (97.30%)	7059200875 (92.81%)	1040146 (0.01%)	3782784987 (49.73%)	7545687506	7347854038 (97.38%)	7010208190 (92.90%)	252178 (0.00%)	3751310586 (49.71%)
Z-S2-CK-3	6858388500	6678670374 (97.38%)	6373821928 (92.93%)	942275 (0.01%)	3366331403 (49.08%)	6816398455	6642358036 (97.45%)	6340686523 (93.02%)	226256 (0.00%)	3344791278 (49.07%)
X-S2-T-1	7636551000	7441856494 (97.45%)	7113835525 (93.16%)	1051007 (0.01%)	3732443644 (48.88%)	7590492113	7403045653 (97.53%)	7078717078 (93.26%)	253402 (0.00%)	3709062727 (48.86%)
X-S2-T-2	7222588200	7041683831 (97.50%)	6707906521 (92.87%)	127589 (0.00%)	3545327649 (49.09%)	7175306627	7000857482 (97.57%)	6670960496 (92.97%)	122534 (0.00%)	3520494342 (49.06%)
X-S2-T-3	7141719900	6964895400 (97.52%)	6637259650 (92.94%)	125765 (0.00%)	3509878802 (49.15%)	7101324089	6930468315 (97.59%)	6606185276 (93.03%)	121045 (0.00%)	3488791631 (49.13%)
Y-S2-T-1	8054530500	7841039022 (97.35%)	7457358667 (92.59%)	142230 (0.00%)	3970262666 (49.29%)	7996637461	7791709977 (97.44%)	7412995893 (92.70%)	137148 (0.00%)	3939557268 (49.27%)
Y-S2-T-2	6424294200	6233938200 (97.04%)	5929058010 (92.29%)	867012 (0.01%)	3198345124 (49.79%)	6372981983	6189922770 (97.13%)	5889149266 (92.41%)	209965 (0.00%)	3172050895 (49.77%)
Y-S2-T-3	6594369900	6416669949 (97.31%)	6124409159 (92.87%)	907313 (0.01%)	3258438981 (49.41%)	6544501891	6373793572 (97.39%)	6085402194 (92.98%)	218134 (0.00%)	3233270069 (49.40%)
Z-S2-T-1	5958458400	5797595440 (97.30%)	5529162197 (92.80%)	810059 (0.01%)	2962949022 (49.73%)	5907843848	5753262789 (97.38%)	5488610499 (92.90%)	196763 (0.00%)	2936755835 (49.71%)
Z-S2-T-2	5947571100	5797283967 (97.47%)	5532850320 (93.03%)	48290 (0.00%)	2926783501 (49.21%)	5914263005	5769483966 (97.55%)	5507763000 (93.13%)	32885 (0.00%)	2909073007 (49.19%)
Z-S2-T-3	6352116600	6176348558 (97.23%)	5888747501 (92.71%)	869197 (0.01%)	3116717250 (49.07%)	6313032591	6143927094 (97.32%)	5859620270 (92.82%)	208288 (0.00%)	3096725986 (49.05%)

Table S3 Statistical results of transcriptome data comparison with reference genomes Total mean the number of reads after ribosome filtration. Unmapped (%) was the number of reads in the unmapped reference genome and its proportion to the effective reads. Unique Mapped (%) refered to the number of reads that were unique to the reference genome and its proportion to the effective reads. Multiple Mapped indicated the number of reads and the proportion of effective reads in multiple sites were mentioned. All mapped was the total number of reads that could be mapped to the genome and its proportion to effective reads.

Sample	Total	Unmapped(%)	Unique Mapped(%)	Multiple Mapped(%)	Total Mapped(%)
X-S1-CK-1	48964534	10733731 (21.92%)	35368786 (72.23%)	2862017 (5.85%)	38230803 (78.08%)
X-S1-CK-2	37491818	8185758 (21.83%)	27101306 (72.29%)	2204754 (5.88%)	29306060 (78.17%)
X-S1-CK-3	39678692	8732217 (22.01%)	28658845 (72.23%)	2287630 (5.77%)	30946475 (77.99%)
Y-S1-CK-1	44851494	9881497 (22.03%)	32467337 (72.39%)	2502660 (5.58%)	34969997 (77.97%)
Y-S1-CK-2	38610270	7991925 (20.70%)	28466833 (73.73%)	2151512 (5.57%)	30618345 (79.30%)
Y-S1-CK-3	40032432	8841134 (22.08%)	28952660 (72.32%)	2238638 (5.59%)	31191298 (77.92%)
Z-S1-CK-1	37798756	8183226 (21.65%)	27551501 (72.89%)	2064029 (5.46%)	29615530 (78.35%)
Z-S1-CK-2	36616972	7857557 (21.46%)	26754009 (73.06%)	2005406 (5.48%)	28759415 (78.54%)
Z-S1-CK-3	42114464	9054793 (21.50%)	30685697 (72.86%)	2373974 (5.64%)	33059671 (78.50%)
X-S1-T-1	36016772	7172984 (19.92%)	26898765 (74.68%)	1945023 (5.40%)	28843788 (80.08%)
X-S1-T-2	42446252	8471942 (19.96%)	31686699 (74.65%)	2287611 (5.39%)	33974310 (80.04%)
X-S1-T-3	48540634	9868647 (20.33%)	36135607 (74.44%)	2536380 (5.23%)	38671987 (79.67%)
Y-S1-T-1	48074710	9341555 (19.43%)	36259465 (75.42%)	2473690 (5.15%)	38733155 (80.57%)
Y-S1-T-2	39045976	7790835 (19.95%)	29288891 (75.01%)	1966250 (5.04%)	31255141 (80.05%)
Y-S1-T-3	39703064	7904863 (19.91%)	29780203 (75.01%)	2017998 (5.08%)	31798201 (80.09%)
Z-S1-T-1	39953850	7990382 (20.00%)	29934744 (74.92%)	2028724 (5.08%)	31963468 (80.00%)
Z-S1-T-2	36453814	7400264 (20.30%)	27176367 (74.55%)	1877183 (5.15%)	29053550 (79.70%)
Z-S1-T-3	45528682	9187191 (20.18%)	33999004 (74.68%)	2342487 (5.15%)	36341491 (79.82%)
X-S2-CK-1	42925098	9613822 (22.40%)	30768340 (71.68%)	2542936 (5.92%)	33311276 (77.60%)
X-S2-CK-2	51281302	11848088 (23.10%)	36346847 (70.88%)	3086367 (6.02%)	39433214 (76.90%)
X-S2-CK-3	50586040	11685753 (23.10%)	35952656 (71.07%)	2947631 (5.83%)	38900287 (76.90%)
Y-S2-CK-1	40310790	8961983 (22.23%)	28966891 (71.86%)	2381916 (5.91%)	31348807 (77.77%)
Y-S2-CK-2	39689620	9159664 (23.08%)	28268982 (71.23%)	2260974 (5.70%)	30529956 (76.92%)
Y-S2-CK-3	44438894	10146304 (22.83%)	31711621 (71.36%)	2580969 (5.81%)	34292590 (77.17%)
Z-S2-CK-1	39908442	9006865 (22.57%)	28597497 (71.66%)	2304080 (5.77%)	30901577 (77.43%)

Z-S2-CK-2	47486636	10645559 (22.42%)	34304665 (72.24%)	2536412 (5.34%)	36841077 (77.58%)
Z-S2-CK-3	44123820	10090763 (22.87%)	31533848 (71.47%)	2499209 (5.66%)	34033057 (77.13%)
X-S2-T-1	48604980	9882995 (20.33%)	36045972 (74.16%)	2676013 (5.51%)	38721985 (79.67%)
X-S2-T-2	47241160	9342982 (19.78%)	35307876 (74.74%)	2590302 (5.48%)	37898178 (80.22%)
X-S2-T-3	46215982	9112225 (19.72%)	34612274 (74.89%)	2491483 (5.39%)	37103757 (80.28%)
Y-S2-T-1	52023688	10300875 (19.80%)	38867042 (74.71%)	2855771 (5.49%)	41722813 (80.20%)
Y-S2-T-2	41518226	9018813 (21.72%)	30337179 (73.07%)	2162234 (5.21%)	32499413 (78.28%)
Y-S2-T-3	42705172	9232173 (21.62%)	31306521 (73.31%)	2166478 (5.07%)	33472999 (78.38%)
Z-S2-T-1	38682032	8276552 (21.40%)	28380695 (73.37%)	2024785 (5.23%)	30405480 (78.60%)
Z-S2-T-2	37907810	7646090 (20.17%)	28178162 (74.33%)	2083558 (5.50%)	30261720 (79.83%)
Z-S2-T-3	40172152	8239373 (20.51%)	29747233 (74.05%)	2185546 (5.44%)	31932779 (79.49%)

Table S4 Information of the metabolic pathways co-enriched in the two growth stages

ID	S1		S2		
	Number	P value	Number	P value	Descrption
ko00500	31	0.0000363	17	0.0302272	Starch and sucrose metabolism
ko01110	139	0.0004274	95	0.0212630	Biosynthesis of secondary metabolites
ko04075	27	0.0066951	23	0.0018027	Plant hormone signal transduction
ko00052	11	0.0078751	9	0.0077552	Galactose metabolism
ko00950	7	0.0202774	9	0.0001740	Isoquinoline alkaloid biosynthesis
ko00905	5	0.0652108	5	0.0206491	Brassinosteroid biosynthesis
